# Photosynthetic Light Harvesting and Thylakoid Organization in a CRISPR/Cas9 Arabidopsis Thaliana LHCB1 Knockout Mutant

**DOI:** 10.3389/fpls.2022.833032

**Published:** 2022-03-07

**Authors:** Hamed Sattari Vayghan, Wojciech J. Nawrocki, Christo Schiphorst, Dimitri Tolleter, Chen Hu, Véronique Douet, Gaëtan Glauser, Giovanni Finazzi, Roberta Croce, Emilie Wientjes, Fiamma Longoni

**Affiliations:** ^1^Laboratory of Plant Physiology, Institute of Biology, University of Neuchâtel, Neuchâtel, Switzerland; ^2^Department of Physics and Astronomy, Faculty of Science, Vrije Universiteit Amsterdam, Amsterdam, Netherlands; ^3^Laboratory of Biophysics, Wageningen University, Wageningen, Netherlands; ^4^Univ. Grenoble Alpes, CNRS, CEA, INRAE, IRIG, LPCV, Grenoble, France; ^5^Neuchâtel Platform of Analytical Chemistry, University of Neuchâtel, Neuchâtel, Switzerland

**Keywords:** photosynthesis, light harvesting, CRISPR/Cas9, LHCII, chloroplast, Arabidopsis

## Abstract

Light absorbed by chlorophylls of Photosystems II and I drives oxygenic photosynthesis. Light-harvesting complexes increase the absorption cross-section of these photosystems. Furthermore, these complexes play a central role in photoprotection by dissipating the excess of absorbed light energy in an inducible and regulated fashion. In higher plants, the main light-harvesting complex is trimeric LHCII. In this work, we used CRISPR/Cas9 to knockout the five genes encoding LHCB1, which is the major component of LHCII. In absence of LHCB1, the accumulation of the other LHCII isoforms was only slightly increased, thereby resulting in chlorophyll loss, leading to a pale green phenotype and growth delay. The Photosystem II absorption cross-section was smaller, while the Photosystem I absorption cross-section was unaffected. This altered the chlorophyll repartition between the two photosystems, favoring Photosystem I excitation. The equilibrium of the photosynthetic electron transport was partially maintained by lower Photosystem I over Photosystem II reaction center ratio and by the dephosphorylation of LHCII and Photosystem II. Loss of LHCB1 altered the thylakoid structure, with less membrane layers per grana stack and reduced grana width. Stable LHCB1 knockout lines allow characterizing the role of this protein in light harvesting and acclimation and pave the way for future *in vivo* mutational analyses of LHCII.

## Introduction

Light is the source of energy for photosynthetic organisms and largely fuels the synthesis of organic molecules in the biosphere. Light excites the chlorophyll (Chl) molecules of the photosynthetic complexes embedded in the thylakoid membrane. The light energy is used by Photosystem II (PSII) for the photo-oxidation of water. The released electrons move from PSII through the electron transport chain (ETC) *via* plastoquinol/plastoquinone (PQ), cytochrome b_6_f (cytb6f), plastocyanin (Pc), to Photosystem I (PSI). Light absorbed by PSI drives the electron transport through ferredoxin to reduce NADP^+^ + H^+^ to NADPH (reviewed in Merchant and Sawaya, 2005). This process is coupled with the generation of a proton gradient between the lumen and the stroma side of the thylakoid membrane, a gradient used by the ATP synthase for the generation of ATP (Boyer, 1993). Light is an inconstant source of energy, and photosynthetic organisms experience shifts in light spectrum and intensity. To cope with these changes and optimize the photosynthetic efficiency, the organization of the pigment-protein complexes must be dynamic (for a recent review, see Rantala et al., 2020). The dynamism of the ETC is also helped by the non-homogeneous distribution of thylakoid membrane proteins. The stacked domains of the thylakoids, called grana, are enriched in PSII, while other domains, composed of non-appressed membranes, are enriched in PSI and are collectively referred to as stroma lamellae. The stacked and unstacked portions of thylakoids are dynamic and contribute to the acclimation to changes in environmental conditions (for a recent review, see Johnson and Wientjes, 2020).

Light is harvested *via* pigment protein complexes embedded in the thylakoid membrane and associated with the two photosystems. The members of these light-harvesting complexes are similar in structure and pigment organization. They can be distinguished by their interactions with the photosystems and spectroscopic properties into (i) LHCI, tightly bound to PSI, (ii) the minor antenna complexes LHCB4 (CP29), LHCB5 (CP26), and LHCB6 (CP24) associated with PSII, and (iii) LHCII, which acts as an antenna of both photosystems (Jansson et al., 1992; Wientjes et al., 2013; Bos et al., 2019; Chukhutsina et al., 2020). LHCI is composed of protein dimers. In higher plants, the most common PSI-LHCI complex contains two heterodimers of LHCI associated with a monomeric PSI core complex (Mazor et al., 2017). These two dimers are composed of different LHCI isoforms: LHCA1 and LHCA4 form one dimer, while LHCA2 and LHCA3 form the second one (Wientjes and Croce, 2011). Each of these isoforms is encoded by a single gene in Arabidopsis (Klimmek et al., 2006). Two additional genes, named *Lhca5* and *Lhca6*, encode close homologs. However, these genes are rarely expressed, and their protein products are only found in sub-stoichiometric amounts compared to PSI reaction centers (Klimmek et al., 2006).

LHCII is the most abundant membrane protein in photosynthetic eukaryotes, accounting for roughly 30% of the thylakoid membrane proteins and harbors half of the total Chl (Peter and Thornber, 1991). The amino acid sequence and the structure of LHCII are highly conserved across plant species. The LHCII, in higher plants, is composed of homo- and heterotrimers of three protein isoforms: LHCB1, LHCB2, and LHCB3 (Caffarri et al., 2004), which share more than 50% sequence identity (reviewed in Barros and Kühlbrandt, 2009; Crepin and Caffarri, 2018). Trimeric LHCII and monomeric antennae LHCB4, LHCB5, and LHCB6 play a central role in the structure of the PSII-LHCII supercomplexes. Two classes of LHCII trimers are present in stable PSII-LHCII supercomplexes. The strongly associated trimers (S-trimers), composed of LHCB1 and LHCB2, interact with the monomeric LHCB4 and LHCB5 isoforms and the inner antenna of PSII. The moderately bound trimers (M-trimers), containing also the LHCB3 isoform, interact only with the monomeric antennae LHCB6 and LHCB4, the latter being connected to the PSII core (for recent reviews, see Croce and Amerongen, 2020; Pan et al., 2020).

An extra pool of LHCII trimers (L-trimers) exists in the thylakoid membranes. These trimers have a loose interaction with PSII and, therefore, are not consistently isolated with the PSII-LHCII complexes (Dekker and Boekema, 2005). The L-trimers have been characterized when associated with PSI in the PSI-LHCI-LHCII complex and found to be composed by LHCB1 and LHCB2 (Kouřil et al., 2005; Galka et al., 2012). The LHCII trimers can thus serve both as antennae of PSII and PSI (Wientjes et al., 2013). The amount of LHCII associated with each photosystem is dynamic and, in part, regulated *via* the phosphorylation of a threonine residue located close to the N-terminus of the LHCII protein (Allen, 1992; Wientjes et al., 2013). In particular, the phosphorylated N-terminus of LHCB2 is capable to interact with PSI (Pan et al., 2018), while LHCB1, in the stable PSI-LHCI-LHCII complex, is mostly non-phosphorylated (Pietrzykowska et al., 2014; Crepin and Caffarri, 2015; Longoni et al., 2015). Despite this difference between LHCB1 and LHCB2, the STN7 kinase (Bellafiore et al., 2005) and the PPH1/TAP38 phosphatase (Pribil et al., 2010; Shapiguzov et al., 2010) regulate the phosphorylation level of both isoforms (Leoni et al., 2013; Longoni et al., 2015). The phosphorylation level of LHCB1 and LHCB2 is linked to the redox state of the PQ pool present in the ETC. In a simplified model, an increased reduction of PQ activates STN7 and thus induces the phosphorylation of its targets (recently reviewed in Longoni and Goldschmidt-Clermont, 2021; Malone et al., 2021). Furthermore, the activity of STN7 is regulated *via* thioredoxins (Ancin et al., 2019) so that the kinase is inactive when plants are exposed to high light intensity, and by the protein amount, for instance, a prolonged over-excitation of PSI by far-red light leads to degradation of STN7 (Willig et al., 2011). While the contribution of LHCB2 and its phosphorylation to the dynamics of the photosynthetic complexes has been described and appears to be conserved across higher plants, the contribution of phosphorylated LHCB1 is less obvious.

LHCB1 is the most abundant isoform composing the trimeric LHCII, based on proteomic analysis a ratio of 7:4:1 for LHCB1:LHCB2:LHCB3 was calculated for Arabidopsis (Galka et al., 2012; Crepin and Caffarri, 2018). In Arabidopsis, there are five genes coding for LHCB1: three genes form a cluster on Chromosome 1 (named *Lhcb1.1*, *Lhcb1.2*, and *Lhcb1.3*), with *Lhcb1.1* and *Lhcb1.3* sharing a common bidirectional promoter (Leutwiler et al., 1986). These three genes encode identical mature proteins with few amino acid differences in the sequence of the transit peptide. The remaining two genes (*Lhcb1.4* and *Lhcb1.5*) form a cluster on Chromosome 2 and code for slightly different mature proteins characterized by three amino acid substitutions compared to the products of the genes on Chromosome 1 (McGrath et al., 1992). Furthermore, the product of *Lhcb1.4* has three extra amino acid substitutions and one deletion in the N-terminal portion of the mature protein that also removes the phosphorylable threonine.

The earlier mutants characterized by a decrease of the LHCII were the mutant of Chl *b* synthesis. These mutants resulted in lower Chl and antenna protein content (Krol et al., 1995; Espineda et al., 1999). However, in these mutants, different antenna isoforms were differentially affected, and previous reports showed an inconsistent effect; further investigations led to the hypothesis that the different isoforms of the light harvesting complex were affected, depending on the growth light intensity (Kim et al., 2009). To investigate the role of the LHCB1 isoform, a more targeted approach was thus required. Almost complete depletion of LHCB1 has been previously obtained by transforming Arabidopsis plants with specific artificial miRNA (*Lhcb1amiRNA*) (Pietrzykowska et al., 2014). This knock-down line revealed that this isoform is contributing *via* STN7-dependent phosphorylation to regulate the photosynthetic electron transport upon light quality changes (Pietrzykowska et al., 2014). However, the study on multiple mutants, lacking both STN7 and PPH1/TAP38, revealed that LHCB1 can also be phosphorylated by the STN7 homolog STN8 (Longoni et al., 2019). The analysis of *Lhcb1amiRNA* showed that LHCB1 is required for the accumulation of trimeric LHCII, and it is important for non-photochemical quenching (NPQ), a collective definition of mechanisms protecting the photosystems from excessive light irradiance (reviewed in Horton and Ruban, 2005; Bassi and Dall’Osto, 2021). Strong reduction of LHCB1, along with LHCB2, was obtained by the introduction of an Lhcb2 antisense gene (Andersson et al., 2003). More recently, by crossing the *Lhcb1amiRNA* with a mutant containing an artificial miRNA targeting LHCB2-coding genes, plants without LHCII trimers and with lower NPQ were obtained (Nicol et al., 2019).

However, there is still no information on the impact of LHCB1 loss on the excitation equilibrium between PSI and PSII, or about the distribution of the photosystems in a “LHCII-depleted” thylakoid membrane. Furthermore, knock-down mutants are prone to changes in gene expression, allowing a leaky accumulation of the target protein, which is not the case for a complete knockout. To investigate the role of LHCB1, we used a CRISPR/Cas9-based approach to mutate simultaneously the five genes encoding this protein. The potential of said approach was previously presented, allowing to reduce the LHCB1 protein amount below the detection limit (Ordon et al., 2020). By targeting identical regions shared between the five *Lhcb1* genes, it was possible to use only two synthetic gRNAs to generate stable mutant lines deprived of LHCB1. We describe how the constitutive and complete loss of LHCB1 affects the antenna organization around the photosystems, its phosphorylation, and the thylakoid structure.

## Materials and Methods

### Plant Material and Mutant Production

For the production of multiple mutant lines, two synthetic gRNAs (sgRNA) were designed based on the Arabidopsis genomic sequence, using the software chop-chop (Montague et al., 2014). The highest ranking sgRNA sequences, targeting multiple *Lhcb1* genes, were further analyzed with E-CRISP (Heigwer et al., 2014). The sgRNA sequences with the highest efficacy score were cloned in a binary vector for the stable transformation of wild type Arabidopsis plants (ecotype: Col0), containing the Cas9 gene under the control of the synthetic EC1 promoter that confers expression in egg cells (Durr et al., 2018). The insertion of two sgRNA was performed following the method described by Xing et al. (2014).

The seed obtained from the transformed plants was germinated on 1/2 MS, containing 33 μg ml^–1^ Hygromycin. After 10 days, resistant plants were transferred to soil. The genomic DNA was extracted from a leaf sample, and a PCR was performed to confirm the presence of the T-DNA insert. The plants containing the T-DNA were led to flowering and the collected seeds germinated on 1/2 MS; the seedlings were screened by Chl *a* fluorescence measurement (detailed below) to detect low NPQ individuals within mixed populations (Supplementary Figure 1). Those individuals were transferred to soil and further analyzed for presence of the T-DNA insert and for the presence of the mutation in the Lhcb1.1–5 genes.

The plants on plates were grown under white light LEDs (120 μmol photons m^–2^s^–1^PAR) with a 16-h-light 8-h-dark daily cycle in a climatic chamber set at 22°C (ARALAB FitoClima 600 PL). The plants in soil were grown under white neon tubes (120 μmol photons m^–2^s^–1^PAR) with a 16-h-light 8-h-dark day cycle in a walk-in climatic chamber set at 22°C. Total Chl extraction was performed on samples composed of three 14 day-old plantlets, which were weighted to measure the total fresh weight, frozen in liquid nitrogen, and grounded to fine powder. The total pigments were extracted in 80% acetone buffered with Tris-HCl pH 7.4 and the Chl *a* and *b* concentration measured by multi-wave length absorbance according to Porra (2002).

### Protein Analysis and Immunodetection

Protein samples were prepared from entire 14 days-old plantlets. Each sample was composed of at least three individuals, the fresh weight recorded, and the plantlets were then flash frozen in liquid nitrogen. The frozen samples were grounded to fine powder using glass beads, mixing in an IvoclarVivadent shaker (Silamat) two times for 10 s. The extraction was performed by homogenizing the sample powder in a lysis buffer, containing 100 mM Tris-HCl, pH 7.8, 2% SDS, 50-mM NaF, and 1 × Protease Inhibitor Cocktail for Plant (Sigma-Aldrich) and then incubating for 30 min at 37°C. The supernatant was clarified by centrifugation and added to a mix composed by 25% of a protein sample, 50% of deionized water, and 25% of a 4x sample buffer (0.2 M Tris/HCl pH 6.8, 0.4 M Dithiotreitol, 8% w/v SDS, 0.4% w/v Bromophenol Blue, 40% v/v Glycerol). The mix was loaded on a Tris-Gly SDS-PAGE 12% acrylamide gel. For the separation of the phosphorylated form of the protein, we used the same gel system to which we added 30 μM Phos-tag (Wako Chemicals) and 60 μM MnCl_2_. The proteins were separated by electrophoresis on the gel and transferred to a nitrocellulose membrane for immuno-detection. The nitrocellulose membranes were blocked with commercial skim-milk 5% (M) in TBS Tween (0.25%), except for the membranes used for the detection of LHCB2, which were blocked with 3% BSA (Applichem) in TBS Triton X-100 (0.1%) to allow the membrane dephosphorylation before the incubation with the primary antibody, detailed in Longoni et al. (2015). After the blocking, the membranes were decorated with primary antibodies for the detection of relevant proteins. The antibodies recognizing the following proteins were obtained from Agrisera: LHCB1 (AS09 522), LHCB2 (AS01 003), LHCB2-P (AS13 2705), LHCB3 (AS01 002), LHCB4 (CP29) (AS04 045), LHCB5 (CP26) (AS01 009), LHCB6 (CP24) (AS04 010), D1 (PsbA) (AS05 084), PsbA-P (AS13 2669), D2 (PsbD) (AS06 146), PsbC (CP43) (AS06 111), PsbB (CP47) (AS04 038), PETC (AS08 330), PsaB (AS10 695), PsaC (AS10 939), PSAD (AS09 461), PSAH (AS06 105), LHCA1 (AS01 005), LHCA2 (AS01 006), LHCA3 (AS01 007), LHCA4 (AS01 008), STN7 (AS16 4098), PPH1 (AS16 4084), STN8 (AS10 1601), ATPC (Agrisera, AS08 312). The antibody recognizing ACT2 (Actin) (A0480) was from Sigma-Aldrich; the anti-PBCP antibody was a gift from Michel Goldschmidt-Clermont. Secondary antibodies (anti-rabbit (Merck, AP132P) or anti-mouse (Sigma, A5278) conjugated with HRP were used for the detection of the primary antibody by enhanced chemiluminescence (ECL); the light signal was recorded using an imager for chemiluminescence (Amersham Imager 600, Amersham Biosciences, Inc.). Band intensity was measured with ImageQuant software (Amersham) and the protein amount estimated based on the signals measured on a dilution scale of the WT sample.

### Thylakoid Preparation and Fractionation

Thylakoid preparation was performed according to Arnold et al. (2014) from adult (4 weeks old) full rosettes. Separation of supercomplexes by blue native polyacrylamide gel electrophoresis was performed as previously described (Järvi et al., 2011), using SERVAGel™ N 3–12, Vertical Native Gels (Serva). The grana and the stroma lamellae fractions were obtained by solubilization of the thylakoid preparation (0.5 mg ml^–1^) with 1% water soluble digitonin (Serva), followed by sequential ultracentrifugation 40,000 × g for 40 min to pellet the grana and 180,000 × g for 90 min to pellet the stroma lamellae.

### Lipid Analysis

The total lipid extraction was performed on samples composed by three 14-days-old plantlets. After measurement of the total fresh weight (FW), the samples were flash frozen in liquid nitrogen and stored at –80°C. The frozen samples were grounded to fine powder using glass beads, mixing in an IvoclarVivadent shaker (Silamat) two times for 10 s. Total lipids were extracted from the powder by adding 10 μl of a tetrahydrofuran: methanol 50:50 (v/v) solution per mg of FW. Plants debris were pelleted by centrifugation (3 min, 14,000 × g); finally, clear supernatant was pipetted to an HPLC vial to perform an ultra-high pressure liquid chromatography separation, followed by atmospheric pressure chemical ionization-quadrupole time-of-flight mass spectrometry (UHPLC-APCI-QTOF-MS) according to Spicher et al. (2016) and Sattari Vayghan et al. (2020). Briefly, the separation was performed on a reverse-phase Acquity BEH C18 column (50 × 2.1 mm, 1.7 μm) maintained at 60°C and analyzed in negative APCI. Mass data were acquired using MassLynx version 4.1 (Waters), and TargetLynx (Waters) was used for processing. Compound identity was determined based on reference standards, which were used for the quantification curves as well (Spicher et al., 2016). Lutein and zeaxanthin, as well as violaxanthin and neoxanthin, could be resolved neither by chromatography nor by mass spectrometry under the conditions employed; therefore, the measured peak corresponds to the sum of both compounds. For the identification of the galactolipids, a commercial DGDG and MGDG mix (Avanti Polar Lipids) was used as a standard, and the linearity of the detection was confirmed as previously reported (Sattari Vayghan et al., 2020).

### Chlorophyll Fluorescence Analysis

Before the measurements, the plants were dark acclimated for at least 15 min. The Chl *a* fluorescence was recorded with MF800 Fluorcam (Photon System Instrument) using a personalized light protocol (Pralon et al., 2020). Briefly, the maximum fluorescence of dark-adapted plants was first measured during a saturating light pulse (F_*M*_). Subsequently, the plants were exposed to steps of increasing blue light intensity. The length of the steps was 1 min for the seedling screening (92; 208; 335; 846; 1,365; and 1,878 μmol photons m^–2^ s^–1^ of PAR intensity), and 5 min for the analyses on adult plants (40, 95, 150, 380, 620, and 850 μmol photons m^–2^ s^–1^ of PAR intensity). At the end of each step, the maximal fluorescence of light-acclimated plants (F_*M*_’) was measured during a saturating light pulse. After each step, the actinic light was turned off; the plants were then exposed for 2 s to far-red light to oxidize the photosynthetic ETC; after that, the recorder fluorescence value was used as F_0_’. To calculate the derived parameters, we used the fluorescence recorded just before the saturating light pulse as F_*S*_. The non-photochemical energy dissipation was measured as NPQ = (F_*M*_–F_*M*_’)/F_*M*_’. PSII quantum yield under light (ΦPSII) was calculated as ΦPSII = (F_*M*_’-F_*S*_)/F_*M*_’ (Papageorgiou and Govindjee, 2004). The fraction of the closed PSII centers (1-qL) was calculated with the following formula: 1-qL = 1–[(F_*M*_’–F_*S*_)/(F_*M*_’–F_0_’)]*(F_0_’/F_*S*_) (Kramer et al., 2004). The Chl fluorescence was also measured with a portable device (Multispeq v2, PhotosynQ, Firmware 2.1), with a modified protocol available at this address: https://photosynq.org/protocols/npq_phi2_from_simple_fluor_light_curve_with_recovery. Due to the LED limitations of this instrument, the time for each light intensity was reduced to 30 s (40, 95, 150, 380, 620, 850 μmol photons m^–2^ s^–1^ of PAR intensity). State transitions and NPQ kinetics were measured on adult plants (4 weeks old) in pots. The state transitions protocol was performed essentially as described using MF800 Fluorcam (Photon System Instrument) (Willig et al., 2011). After 30 min of dark adaptation and the measure of the Fm, 25 μmol photons m^–2^ s^–1^ of red light were used to induce State 2 and red light supplemented with far red light to induce State 1. Each light condition lasted 10 min. The maximal fluorescence was recorded during a saturating light pulse (500 ms) at the end of each light phase. The qT was calculated as (FmSt1-FmSt2)/FmSt1. The NPQ induction and the relaxation curve were measured with the same MF800 Fluorcam, using a protocol adapted from Nicol et al. (2019). Briefly, after the measurement of the Fm in plants dark adapted for 1 h, the plants were exposed to 10 min of 1,000 μmol photons m^–2^ s^–1^ actinic light (837 μmol photons m^–2^ s^–1^ blue and 165 μmol photons m^–2^ s^–1^ red light). The light was turned off, allowing the relaxation of the NPQ for 10 min. During the light and dark periods, the maximal fluorescence was measured with saturating light pulses (500 ms, 100% intensity) at intervals of 20 s. For the second light induction, the same light, dark, and measurement protocol was repeated.

Rapid Chl *a* fluorescence induction was measured on detached leaves with the Plant Efficiency Analyzer (M-PEA 2; Hansatech Ltd.). The following protocol was used: after an initial saturating pulse (3,000 μmol photons m^–2^ s^–1^, 700 ms, red light, dominant λ625 nm), the same pulse was repeated after sequentially longer dark intervals (0.05, 4, 8, 12, 16, 20, and 24 s) for a total of eight pulses. After the eighth pulse, far-red light (20%) was turned on, and the sample was submitted to a second series of saturating pulses separated by increasing far-red light intervals (0.05, 0.1, 0.2, 0.4, 0.8, and 1.6 s). For each pulse, the fast Chl fluorescence curve was extrapolated by the M-PEA2 software (Hansatech Ltd.). The variable fluorescence at 3 ms (Vj) was analyzed by plotting as a function of the time between pulses.

To assess the functional antenna size of PSII, fluorescence induction measurements upon a transition from darkness to low light were performed on DCMU [3-(3,4-dichlorophenyl)-1,1-dimethylurea]-infiltrated leaves according to Nicol et al. (2019). In brief, in the presence of DCMU, the rise to the fluorescence maximum (F_*M*_) represents a single, stable charge separation in PSII, the rate of the latter being directly proportional to the PSII absorption cross-section at a given light intensity. The reciprocal of the integrated area above the induction curve yields the maximal initial rate of PSII (when all PSIIs are open) (Papageorgiou and Govindjee, 2004).

FLIM measurements were performed on whole leaves incubated with DCMU with the same setup as the confocal images (see section “Confocal Microscopy”), except that it was coupled to a PicoHarp 300 time-correlated single photon counting (TCSPC) module (PicoQuant, Berlin, Germany) as described in Wientjes et al. (2017). The samples were excited with 633-nm light (40-MHz rep. rate), and fluorescence emission was detected at 710–750 nm. Each image was recorded for 1 min. The total image size was 116 * 116 μm, with 128*128 pixels. The time step for the TCSPC detection was 32 ps/channel. Decay traces were fitted with three exponentials *F*(t) = a1*e^ (–t/τ1) + a2*e^(–t/τ2) + a3*e^(–t/τ3) with (a) the amplitude and (τ) the lifetimes (τ1 = 100 ps, τ2 = 900 ps, and τ3 = 2 ns) using the FLIMfit software tool (Warren et al., 2013). Based on the amplitude of the PSI lifetime (100 ps), the ratio of Chls associated with PSI compared to PSII can be calculated according to PSI/(PSI + PSII) = c*a1/(1-a1 + c*a1) with c being a previously determined correction factor for this specific setup (*c* = 0.49) (Wientjes et al., 2017).

### Absorption Spectroscopy *in vivo*

For the calculation of the ratio of CEF over LEF, we used the protocol described in Kramer et al. (2021). Briefly, the plants were grown under long-day conditions and initially dark incubated for 60 min and then 5 min before transfer into actinic light with 630-μmol photons m^–2^ s^–1^ PAR for light acclimation. After 10, 20, 40, 60 s or 5 min of illumination, LEF and CEF were measured by following the relaxation kinetics of the carotenoid electrochromic bandshift at 520 nm (corrected for the signal at 546 nm) using a JTS-10 spectrophotometer (Biologic, France). The ECS spectral change is a shift in the pigment absorption bands, which is linearly correlated with the light-induced generation of a membrane potential across the thylakoid membranes (Bailleul et al., 2010). Under steady-state continuous illumination, the ECS signal stems from transmembrane potential generation by PSII, the cytb6f complex, and PSI and from transmembrane potential dissipation by the ATP synthase CF_0_-F_1_. When light is switched off, reaction centers’ activity stops immediately, while ATPase and the cytb6f complex activities remain (transiently) unchanged. Therefore, the initial rate of ECS decay is proportional to the rate of PSI and PSII photochemistry (i.e., to the rate of “total” electron flow). This can be calculated by dividing this rate (expressed as –ΔI/I per unit of time) by the amplitude of the ECS signal (again expressed as –ΔI/I) induced by the transfer of one charge across the membrane (e.g., one PSI turnover). The rate of CEF can be evaluated using the same approach under conditions where PSI only is excited by exposure to saturating far red light (λ > 720 nm) for enough time to be at the steady state (5 min in our condition). Results were expressed as electrons^–1^ s^–1^ and estimated from the amplitude of the electrochromic shift signal upon excitation with a saturating single turnover flash [5 ns Nd:YAG laser flash, Continuum Minilite, 532-nm flash exciting 4-(dicyanomethylene)-2-methyl-6-(p-dimethylaminostyryl)-4H-pyran (DCM), emission peak at 630 nm]. Total electron flow was measured following a pulse of actinic light (λ = 640 ± 20 nm FWHM) at 1,100 μmol photons m^–2^ s^–1^, while CEF was measured with a pulse of far-red light at the maximum setting (estimated as 1,400 μmol photons m^–2^ s^–1^ by the manufacturer).

Functional antenna size of PSII and PSI was assessed using ECS as described in Hu et al. (2021). In brief, similar to the light-to-dark transition described above, the initial slope of the ECS signal during the onset of light is directly proportional to the absorption-limited, maximal rate of charge separation by fully open PSII + PSI. Addition of DCMU and hydroxylamine by vacuum infiltration of the leaf inhibited PSII activity (systematically verified with fluorescence measurements), allowing to separately quantify the rates of both PSII and PSI. The rates in e^–^ s^–1^ PSI^–1^ (or e^–^ s^–1^ PSII^–1^) were obtained by dividing the slope by the ECS signal obtained 140 μs after a saturating, single-turnover laser flash (see above), corresponding to 1 charge separation PS^–1^.

### Confocal Microscopy

Confocal images were recorded on an (inverted) confocal Leica TCS SP8 system equipped with a 63 × 1.20 NA water immersion objective. Chloroplasts were freshly isolated, and Chls were excited at 633 nm with a pulsed laser (40 MHz) and an intensity of 100 nW. The fluorescence was detected at 650–680 nm (PSII-maximum) and 710–750 nm (PSI-maximum) with internal hybrid detectors. The total image size was 9.2 * 9.2 μm, with 128*128 pixels.

### Transmission Electron Microscopy

Samples preparation and analysis were performed as previously described (Martinis et al., 2013), with minor modifications. Leaves from 14-day-old WT and L1ko plants were fixed in a fixative buffer [5% (W/V) glutaraldehyde and 4% (W/V) formaldehyde in a 100-mM phosphate buffer (pH 6.8)] overnight at 4°C, rinsed several times in a phosphate buffer, and post fixed for 2 h with 1% (W/V) osmium tetroxide in a phosphate buffer at 20°C. After two further washing steps in a phosphate buffer and distilled water, the samples were dehydrated in ethanol and embedded in Spurr’s low-viscosity resin (Polyscience). Ultrathin sections of 50–70 nm were cut with a diamond knife (Ultracut-E microtome- Reihtert-Jung), mounted on uncoated copper grids. The sections were post stained with Uranyless and Reynold’s lead citrate stain (Delta Microscopies). Sections were observed with a Philips CM 100 transmission electron microscope operating at 60 kV (Philips Electron Optics BV, Eindhoven, the Netherlands).

### Statistical Analysis

The normal distribution of the residuals of each data set was tested before any other statistical analysis. If this assumption was met, an ANOVA model was utilized; otherwise, a Kruskal–Wallis rank sum test was performed. If the results were significant, we used *post hoc* Student’s *t*-test for multiple comparisons. The reported *p*-values were obtained with the latter. The effect of genotypes on the correlation between the number of stacks per grana or grana width was tested using analyses of covariance, with number of stacks as a response variable, and the genotypes by grana, or by grana width as factors. We used a generalized linear model (GLM) with Poisson distribution for assessing the numbers of stacks, and a linear model for grana width. The calculations were performed with RStudio (Version 1.2.5019 RStudioInc).

## Results

### Production of Lines Deprived of LHCB1

**FIGURE 1:**
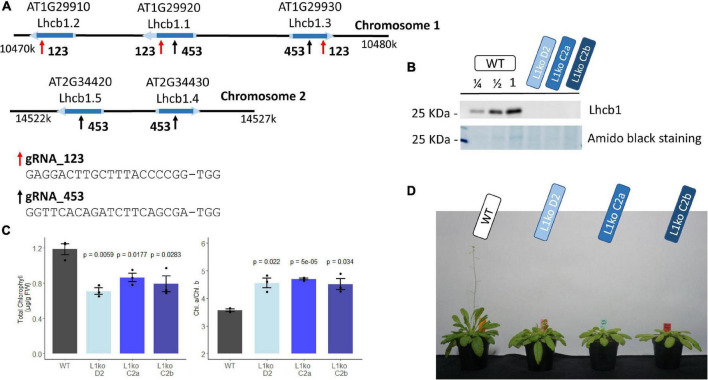
Generation of total knock-out lines for the five genes encoding LHCB1. **(A)** Schematic representation of the Lhcb1 gene clusters, showing the regions targeted by the two sgRNAs; the sequence of the sgRNAs is reported below. **(B)** Protein accumulation of the produced mutant lines; the representative immunoblot shows the detection of LHCB1 in a dilution series of WT total protein extract and in the L1ko mutants. Below, the staining of the membrane with amidoblack shows the differential band at 25 KDa that corresponds to the LHCII protein. **(C)** Total Chl accumulation normalized over the plant fresh weight, left, and the Chl *a*/*b* ratio, right, in WT and in the three L1ko mutant lines. The error bar indicates the standard error, and individual replicates are plotted as points (*n* = 3) above each L1ko line are reported the *p*-value of a Student’s *t*-test comparison with WT. **(D)** The representative flowering phenotype of adult plants of the WT and the three independent L1ko lines.

The Arabidopsis genome contains five genes encoding *quasi*-identical LHCB1 proteins. These genes are organized in two close regions. Chromosome 1 contains *Lhcb1.1* (AT1G29920), *Lhcb1.2* (AT1G29910), and *Lhcb1.3* (AT1G29930). The remaining two, *Lhcb1.4* (AT2G34430) and *Lhcb1.5* (AT2G34420), are located on Chromosome 2. Due to their close proximity, it would not be possible to obtain multiple insertional mutants by crossing. A CRISPR/Cas9 approach was used to insert simultaneously a mutation in all the genes. As these genes have a largely identical nucleotide sequence, it was possible to design two gRNAs targeting all of them (Figure 1A). Five plants were transformed with the construct containing the two gRNAs and the gene encoding the Cas9 endonuclease. From each plant, we obtained two to four resistant progenies (T1 generation), which were screened for NPQ, as a loss of *Lhcb1* should lower the photoprotective capacity (Pietrzykowska et al., 2014; Supplementary Figure 1). The candidates were self-crossed to obtain stable T2 lines. To characterize the nature of the mutation, we sequenced each targeted gene of two independently selected T2 lines. In the line named D2, five different knockout mutations were identified: *Lhcb1.2* (AT1G29910), which has a single nucleotide deletion in the codon for Tyr 191; *Lhcb1.1* (AT1G29920), containing a single nucleotide insertion in the codon for Ser 140; *Lhcb1.3* (AT1G29930), with a single nucleotide insertion in the Pro 192 codon; in *Lhcb1.4* (AT2G34430), a single nucleotide insertion occurred in the codon for Ser 139, and, finally, *Lhcb1.5* (AT2G34420), harboring a large deletion from the Ser 138 codon to the Pro190 codon. A second line, named C2, displayed a deletion/rearrangement between the genes *Lhcb1.2* (AT1G29910) and *Lhcb1.1* (AT1G29920) that made impossible to amplify the gene sequences plus a single nucleotide deletion in the codon for Pro 192 of *Lhcb1.3* (AT1G29930). The mutations on *Lhcb1.4* (AT2G34430), single nucleotide insertion in Ser 139 codon, and *Lhcb1.5* (AT2G34420), single nucleotide insertion in Ser 138 codon, were heterozygous in the T2 line. Therefore, this line was further self-crossed to obtain two T3 lines: one with no mutation in these two genes (C2a) and one carrying the mutations in both genes (C2b). However, even in the presence of non-mutated Lhcb1 genes on Chromosome 2, in both C2-derived lines, there was no detectable LHCB1 protein nor a detectable band by total protein staining at the LHCII level (Figure 1B). We will refer to the multiple LHCB1 mutants, lacking any detectable LHCB1 protein, as L1ko mutants.

The L1ko mutant plants have a pale green phenotype, with an average Chl per fresh weight content corresponding to 66 ± 6% of the WT (Figure 1C). Consistent with the notion that LHCII is enriched in Chl *b*, this loss was uneven between Chl *a* and *b*, and caused an increase in the Chl *a*/*b* ratio in L1ko lines compared to the WT (Figure 1C). Furthermore, the plant growth was also slower, resulting in a smaller rosette and a delay in flowering (Figure 1D).

To compare the generated mutant lines, we analyzed their photosynthetic performance in a range of increasing light intensities. This analysis revealed that there was no statistically significant difference between the three L1ko lines in terms of NPQ, quantum yield of the photosystem II (ΦPSII), and the fraction of the closed PSII reaction centers (1-qL) (Supplementary Figure 2).

In summary, the CRISPR/Cas9 system based on two sgRNAs allowed for the simultaneous mutation of multiple LHCB1-encoding genes. Multiple stable lines produced are characterized by an undetectable amount of the LHCB1 protein and a pale phenotype. All the L1ko lines have a similar photosynthetic activity profile, showing that the loss of LHCB1 correlates with the photosynthetic defect, independently of the presence of non-mutated *Lhcb1.4* and *Lhcb1.5* genes.

### A Profile of Thylakoid Proteins in L1ko

**FIGURE 2:**
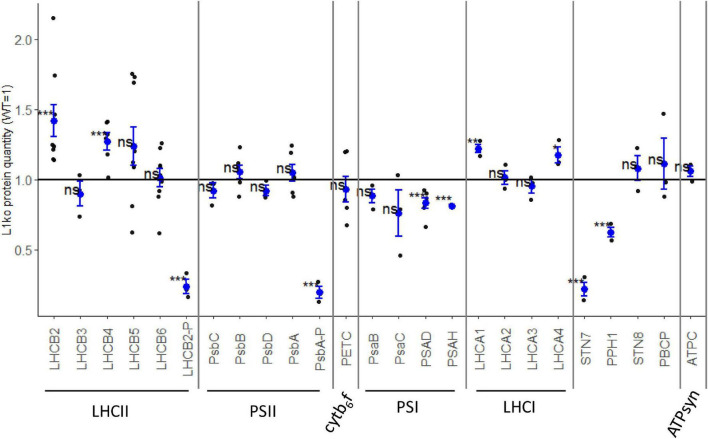
Few thylakoid proteins are affected by the loss of LHCB1. The accumulation of proteins was assessed in 14-days-old seedlings of WT and L1ko lines by immunoblotting. The relative protein accumulation, normalized over the actin, was measured compared to the WT level. Each point represents the quantification of the relevant protein in an individual biological replicate; we analyzed one sample per genotype for proteins with low variation between lines and two or three samples for proteins with larger variation. The blue dot represents the average and the blue bars the standard error of the distribution. The proteins were divided into the complexes to which they are associated (LHCII, PSII, cytb6f, PSI, LHCI, and ATPsyn). The accumulation kinases and phosphatase mainly involved in the regulation of the phosphorylation of LHCII and PSII were also measured. The measured levels were compared to the average of 1, corresponding to the protein accumulation in WT extracts *via* a Student’s *t*-test; asterisks indicate *p*-value thresholds (0.0001 < *p* < 0.001 “***”, 0.001 < *p* < 0.05 “**”, 0.05 < *p* < 0.10 “*”, 0.10 < *p* “ns”) (see Supplementary Figures 3A,B).

It was previously reported that the removal of LHCB1 by miRNA has a limited impact on the accumulation of other thylakoid proteins, with the exception of LHCB2 (Pietrzykowska et al., 2014). We investigated if the protein accumulation (relative to fresh weight) was modified in the stable L1ko lines compared to WT (Supplementary Figure 3). Figure 2 reports the average of the three independent lines D2, C2a, and C2b to highlight differences in protein accumulation due to the loss of the LHCB1 protein, independently of the genetic background. In the L1ko lines, we observed a significant increase in the accumulation of LHCB2 (1.42 ± 0.33-fold change) and a small one in LHCB4 (1.27 ± 0.15-fold change) compared to WT. There was, however, little to no detectable difference in the accumulation of LHCB5 (1.24 ± 0.40-fold change), LHCB6 (1.01 ± 0.19-fold change) or LHCB3 (0.89 ± 0.15-fold change) (Figure 2). Taken together, these results show that the loss of LHCB1 did not cause any large—and thus detectable by immunoblotting—compensatory changes at the protein level of the minor LHCBs. The larger variation is at the level of LHCB2, whose accumulation is slightly increased in a fashion similar to the previous report on the *amiLhcb1* line (Pietrzykowska et al., 2014). We also assessed the accumulation of the subunits of the core of PSII (PsbA to D); these proteins did not display any significant difference when compared to WT levels of accumulation (Figure 2).

As for the subunits of PSI, we could not detect a significant difference in the core subunits PsaB and PsaC despite a general tendency toward a lower level of accumulation in L1ko lines compared to WT (0.88 ± 0.09 and 0.76 ± 0.29-fold change, respectively). The decrease was instead significant for the peripheral subunits PsaD (0.83 ± 0.09-fold change) and PsaH (0.81 ± 0.01-fold change), as their signal was consistently lower in L1ko lines compared to the WT (Figure 2). It has to be highlighted that this difference between the core and peripheral subunits of PSI may stem from the semi-quantitative nature of the immunodetection rather than of a physiological difference. Conversely, two of the four proteins composing the PSI antenna, LHCA1 and LHCA4, appeared to be slightly, but significantly, more abundant in L1ko lines compared to WT (1.22 ± 0.06 and 1.17 ± 0.10-fold change, respectively), while no significant difference was observed for LHCA2 and LHCA3. We measured the level of PETC protein as a proxy for the cytb6f complex amount; this protein did not show any significant difference in accumulation between L1ko lines and the WT. Similarly, there was no difference in the accumulation level of ATPC, used as a proxy for the ATP synthase complex. The phosphorylation level of LHCB2 and of the two PSII subunits, PsbA and PsbC, was clearly lower in L1ko lines compared to the WT (Figure 2 and Supplementary Figure 4), as assessed by antibodies recognizing the phosphorylated form of these two proteins and by phos-tag gels.

Following on the altered phosphorylation of LHCB2 and PSII in L1ko, we assessed the accumulation of the relevant kinases and phosphatases. The accumulation of STN7 (i.e., the kinase mainly involved in LHCII phosphorylation) was strikingly lower in the L1ko lines compared to the WT (0.22 ± 0.08-fold change). This was also the case for the phosphatase TAP38/PPH1, which counters the activity of STN7, which was less accumulated in L1ko lines compared to the WT (0.62 ± 0.06-fold change). Conversely, the second kinase/phosphatase pair, consisting of the thylakoid kinase STN8 and its counteracting phosphatase PBCP, did not show any significant difference in accumulation in L1ko compared to WT.

Taken together, these results show that LHCB1 mutation caused a minor change in the composition of the photosynthetic proteins. Protein analysis suggests that most of the compensatory responses occur at the level of ETC proteins activity and interaction, notably, by changing the phosphorylation level. As the potential difference in the PSII and PSI relative accumulation and antenna size between L1ko and WT should be rather minor, the immunodetection based on chemio-luminescence is not sufficient to assess it conclusively. This prompted us to pursue further biophysical analysis.

### Decrease of PSII Antenna Size in L1ko Is Partially Compensated by the Increased PSII/PSI Ratio

**FIGURE 3:**
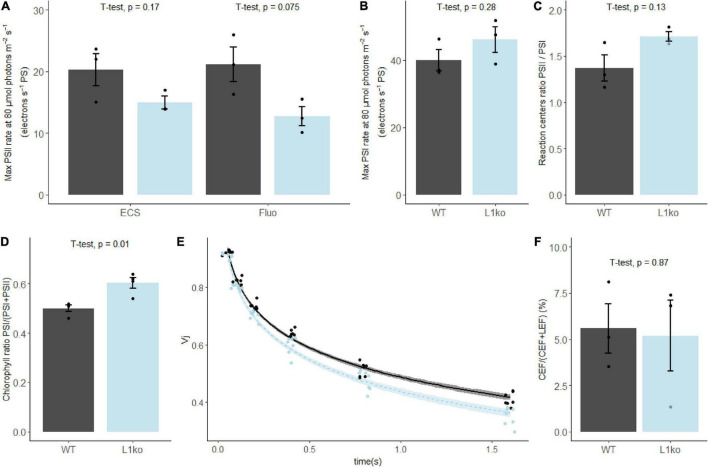
In plants lacking LHCB1, the chlorophyll repartition is shifted toward PSI. **(A)** The maximal photochemical rate of PSII, measured by electrochromic shift (ECS) and fluorescence (fluo) and **(B)** PSI measured by ECS. **(C)** The ratio of PSI/PSII reaction centers measured by a single turnover flash. **(D)** The average PSI/(PSI + PSII) Chl ratio determined from the 100 ps component of the fluorescence decay measured in fully expanded leaves, excitation wavelength at 633 nm and fluorescence emission at 710–750 nm (see Supplementary Figure 5). **(E)** Fluorescence value measured at 3 ms during a 700 ms saturating pulse normalized over the maximal fluorescence (VJ), upon sequential incubations of the sample with far-red light with increasing time intervals. The points were fitted with a local regression algorithm based on a logarithmic function to visually represent the data distribution in function of the time with a continuous error estimate (black continuous line WT, light blue L1ko). The standard error of the interpolation is shown as a gray area around the curves. **(F)** Contribution of CEF to the total electron flow in WT and L1ko expressed as a percentage of the electron flow. Individual biological replicates are plotted as points, the histogram shows the average, and the error bars represent the standard error (*n* = 3, *n* = 4 for **E**). The averages of the distributions were compared with Student’s *t*-test, and the *p*-value of the comparison between WT and L1ko reported above the bars.

LHCII antenna has the important role to equilibrate the excitation between the two photosystems. This dynamic equilibrium allows the photosynthetic apparatus to optimize the linear electron transport and to respond to variations in light intensity and quality (Allen, 1992; Ballottari et al., 2012). As the L1ko mutants showed an important loss of the LHCII complex (Supplementary Figure 5), we investigated the ratio of the photosystems and their relative antenna size. To assess which proportion of the antenna was connected to the two photosystems in the mutant background, the photochemical rate of both photosystems was measured by electrochromic shift (ECS) and fluorescence rise time. This analysis showed that the PSII maximal photochemical rate at 80 μmol photons m^–2^ s^–1^ was 25% lower in L1ko on a per-photosystem basis (14.95 ± 1.78 electrons⋅s^–1^⋅PS^–1^) compared to WT (20.25 ± 4.55 electrons⋅s^–1^⋅PS^–1^). Note that the absolute value of maximal PSII rate at 80 μmol⋅photons⋅m^−2^⋅s^−1^ obtained with both fluorescence- and ECS-based methods is identical (Figure 3A). The PSI maximal photochemical rate was unchanged (WT, 39.98 ± 5.48 electrons⋅s^–1^⋅PS^–1^; L1ko, 46.14 ± 6.56 electrons⋅s^–1^⋅PS^–1^) (Figure 3B). The relative amount of the two photosystems partially compensated for the difference in the photochemical rate. The PSII/PSI reaction centers ratio was 1.37 ± 0.25 in the WT and 1.71 ± 0.09 in L1ko (Figure 3C). By multiplying the average photochemical rate measured for PSI and PSII by the average PSII/PSI reaction centers ratio, we can estimate a global PSI/(PSI + PSII) Chl ratio of 0.59 for WT and 0.64 for L1ko. This difference in the global Chl ratio between the two photosystems in WT and L1ko was found also by analyzing the fluorescence decay. The calculated PSI/(PSI + PSII) Chl ratio was of 0.52 ± 0.01 for WT and 0.60 ± 0.01 in L1ko (Figure 3D and Supplementary Figure 6). The difference between PSI and PSII Chls is also reflected in the rate of Q_*B*_ oxidation as estimated by the Vj parameter, which is the relative fluorescence measured at 3 ms during a saturating light pulse, normalized over the maximal fluorescence (Toth et al., 2007). In fact, in a leaf exposed to far-red light, the decrease of the Vj value over time is faster in L1ko (the Vj trend over time, –0.303) compared to the WT (–0.281) (Figure 3E). The same does not occur in the dark; in this situation, the slope of the Vj decrease over time is comparable between the two genotypes: –0.149 for L1ko and –0.147 for WT (Supplementary Figure 7). This supports the idea that the faster oxidation of the photoactive plastoquinone is due to the higher activity of PSI over PSII and not to an increased activity of the non-photochemical plastid oxidase (Nawrocki et al., 2015). The different distributions in the excitation of the photosystems did not result in a change in the proportion of the two main photosynthetic electron transport routes; in fact, the proportion of the cyclic electron flow (CEF) around PSI to the linear electron flow (LEF) was comparable between L1ko and WT (Figure 3F).

**FIGURE 4:**
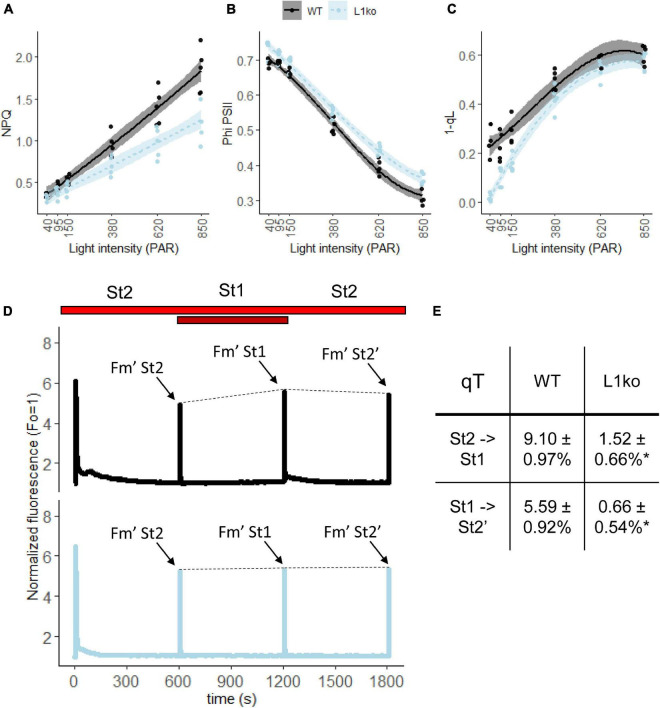
Loss of LHCB1 alters the equilibrium of the LEF between PSII and PSI. The LEF was monitored by room temperature Chl fluorescence on adult WT (black lines) and L1ko (light blue lines) plants. The plants were exposed to a range of increasing light intensities, each lasting 5 min. **(A)** Non-photochemical quenching (NPQ), **(B)** quantum efficiency of PSII, referred as ΦPSII in the main text. **(C)** Fraction of closed PSII centers estimated by the 1 qL parameter. Each experimental point is displayed as a dot. The measured points were fitted with a second-degree polynomial function for NPQ, a third-degree polynomial for ΦPSII and 1-qL to visually represent the data distribution and the standard error for WT (the black line and the gray area) and L1ko (the blue dotted line and the light blue area). *Post hoc* analysis of the models shows that there is a significant difference between L1ko and WT (*p* < 0.001). **(D)** Chl *a* fluorescence traces measured during shifts from State 2 (St2) to State 1 (St1), light and back. The line represents the average fluorescence and the area the standard deviation (*n* = 3) for WT (upper, the black line) and L1ko (lower, the blue line). The bars at the top indicate illumination with red (shown in red) and far-red (dark red) light. The dashed line connecting the Fm’ peaks was added to highlight the state transitions. **(E)** qT values calculated as (Fm’St1-Fm’St2)/Fm’St2 ± SD (*n* = 3), the peaks used for the calculation are highlighted in **(D)**, and the values indicated with an * are statistically different (*p* < 0.01).

The faster PQ oxidation in far-red and the higher PSI/(PSI + PSII) Chl ratio measured in L1ko plants should result in a larger ΦPSII and open PSII reaction centers under light-limiting conditions. To assess that, we performed a follow-up experiment using adult plants. The room temperature Chl *a* fluorescence transient on WT and L1ko exposed to a range of increasing light intensities (from 40 to 850 μmol⋅photons⋅m^–2^⋅s^–1^ PAR) was measured with a fluorescence camera as well as a portable device. Consistently, with our hypothesis, the ΦPSII resulted to be higher, especially in the initial range of light intensities used in L1ko compared to WT. The higher quantum yield in L1ko correlated with a larger fraction of open PSII reaction centers estimated by the 1-qL parameter (Figure 4). Furthermore, in agreement with previous results during the screening for L1ko mutants and the literature (Pietrzykowska et al., 2014; Nicol et al., 2019), the NPQ amplitude was lower in L1ko compared to WT (Figure 4). The same trends for NPQ and ΦPSII parameters were confirmed by a second measurement with the portable device (Supplementary Figure 8). The measurement of the state transitions by fluorescence in WT and L1ko is in agreement with the previous observation (Figure 4). In L1ko, the state transitions amplitude (qT) is reduced compared to WT. The change in the PSI/(PSI + PSII) Chl ratio could also be responsible for the lack of the transient reduction of the ETC that is visible in the WT as an increase of the Chl *a* fluorescence upon a switch from State 1 to State 2 light (Figure 4).

**FIGURE 5:**
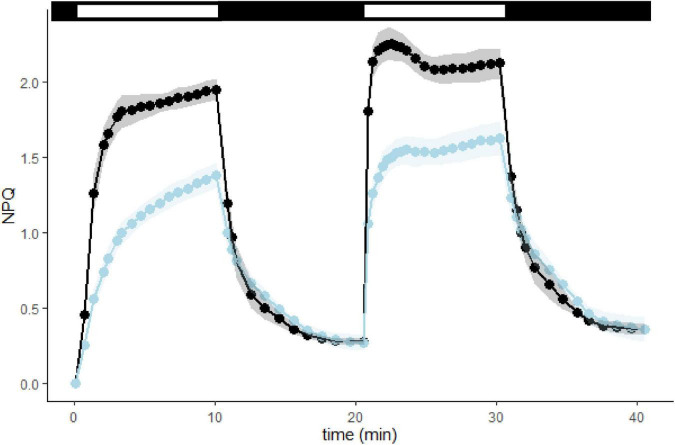
L1ko is affected in the rapidly reversible component of NPQ (qE). NPQ kinetics with two periods of light induction, both at 1,000 μmol photons m^– 2^ s^– 1^, measured in WT and L1ko adult plants. The top bar shows the light exposition periods (white bars) and the dark relaxation (black bars). The points and the lines correspond to the average NPQ value recorded for each time from five biological replicates. The lines and areas show a continuous estimate of the average and the standard deviation for WT (the black line and the gray area) and L1ko (the blue line and the light blue area). The corresponding fluorescence traces are shown in Supplementary Figure 9.

However, the loss of qT is not enough to explain the difference in NPQ observed between WT and L1ko as, at higher light intensities, the NPQ governs the redox state of the ETC. We, therefore, conducted a follow-up analysis measuring the NPQ induction and relaxation kinetics (Figure 5 and Supplementary Figure 9). We observed that L1ko had a significantly lower induction of the rapidly reversible component of NPQ (qE). In fact, at the end of the light phase, the total NPQ is still a significantly lower in L1ko (1.38 ± 0.08) compared to WT (1.95 ± 0.07), but, after 5 min of dark relaxation, both L1ko (0.27 ± 0.05) and WT (0.27 ± 0.02) had comparable levels of NPQ. The second phase of NPQ induction after 10 min of dark relaxation shows a faster induction in both L1ko and WT. This is due to the persistence of the Zeaxanthin-dependent NPQ component due to the kinetics of the zeaxanthin conversion back to violaxanthin in the dark (Siefermann and Yamamoto, 1975).

In conclusion, the loss of LHCB1 has a larger impact on the photochemical rate of PSII than on that of PSI. This imbalance is partly compensated by an increase of the relative PSII/PSI reaction centers ratio. However, this does not allow L1ko plants to reach the same photosynthetic equilibrium of the WT. The photoprotection is also impaired in L1ko, as seen by a lower extent of qE and qT induction.

### Loss of LHCB1 Alters the Thylakoid Organization

**FIGURE 6:**
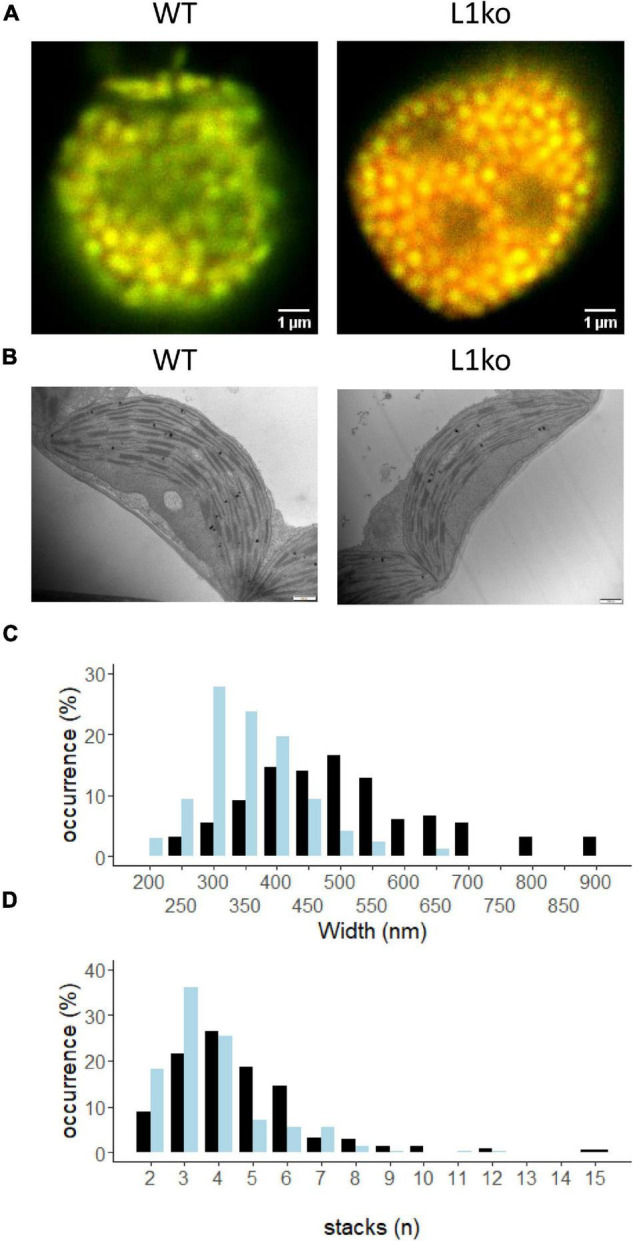
LHCB1 mutation affects the thylakoid structure. **(A)** Confocal microscopy on isolated chloroplasts of WT and L1ko. Excitation at 633 nm and detection at 650–680 nm are shown in green (PSII-maximum), while 710–750 nm detection in red (PSI-maximum). Red channel intensity has been increased 10 times in comparison to the green channel to compensate for decreased sensitivity of the detector and decreased fluorescence emission in the far-red region (see Supplementary Figure 10). **(B)** Representative images of chloroplasts from WT and L1ko leaves obtained by transmission electron microscopy. The scale bar is 500 nm (see Supplementary Figure 12). **(C)** Distribution of the grana width in WT and L1ko based on the measurement of 164 and 173 grana for WT and L1ko, respectively. **(D)** Distribution of the grana stacks divided by the number of layers based on the analysis of 215 and 241 grana for WT and L1ko, respectively. The chloroplast images used for the analysis were obtained from two independent biological samples. The statistical analysis of the grana stack number and width distributions is presented in the text.

The trimeric LHCII is a large component of the thylakoid membrane. Consequently, a change in the shape and organization of the thylakoid membrane is expected upon removing LHCB1, which constitutes a large portion of LHCII. Therefore, the chloroplast ultrastructure was investigated in the mutant lines by transmission electron microscopy (TEM) and confocal microscopy. While confocal microscopy allows to observe the overall chloroplasts topology and the distribution of the photosystems, the TEM images allow to observe the fine structure of the thylakoids (Figure 6). Confocal microscopy images of isolated chloroplasts were acquired using two detection ranges to preferentially detect either the emission of PSII (650–680 nm, green), or of PSI (710–750 nm, red) (Figure 6A and Supplementary Figure 10). These images showed relatively more Chl connected to PSI and smaller grana in L1ko compared to the WT (yellow areas in Figure 6A and Supplementary Figure 11). Since the spatial resolution of confocal microscopy is too low, TEM images were used to measure the grana widths and number of grana layers per stack (Figure 6B and Supplementary Figure 12). These analyses showed that the loss of LHCB1 leads to fewer stacks per grana on average (3.75) compared to the WT (4.57). Covariance analysis showed a significant negative correlation between the number of grana and the number of stacks for both the WT and L1ko (*z*-value = –13.8, *p* < 0.001), but such correlation was different across genotypes (interaction term: *z*-value = 3.12, *p* = 0.002), which results in an average of 21% less stacks per grana in L1ko compared to WT (genotype effect; *z*-value = –3.4, *p* = 0.001). Concerning grana width, the variance analysis shows a significant difference between the genotypes (*p* < 0.001), with an average grana width for the WT of 500 nm and an average width of 356 nm for the L1ko mutant. Consistently, with the observation of the reduced grana width and stacks number, upon separation of the grana stacks from total thylakoids by digitonin, the grana fraction from WT thylakoids contained 66 ± 4% of the total Chl, while, in L1ko, this percentage was reduced to 40 ± 14% (Supplementary Figure 13).

To assess whether the change in the LHCII content had an impact on the lipid portion of the thylakoid membrane, we measured the total content of the two main classes of galactolipids, monogalactosyldiacylglycerols (MGDGs), and digalactosyldiacylglycerols (DGDGs). This analysis showed that the total galactolipid content, normalized over the fresh weight, was unchanged between the L1ko lines and the WT. In addition, the relative abundance of the different molecules sorted by the degree of saturation and length of the acyl chains was comparable between the L1ko lines and the WT (Supplementary Figure 14). The main prenyl lipids found in the thylakoid membrane were also accumulated to the same level in L1ko and WT; only the xanthophylls, which are mostly associated with LHCII proteins (i.e., violaxanthin, neoxanthin, zeaxanthin, and lutein) were clearly less abundant in the L1ko lines compared to the WT. Conversely, there was no change in β-carotene content (Supplementary Figure 14).

## Discussion

### Three of the Five Genes Encoding LHCB1 Are the Major Contributors to the Protein Accumulation

Stable multiple mutant lines are fundamental to study the function and the role of the major components of the LHCII. The two major isoforms, LHCB1 and LHCB2, are coded by multiple genes; therefore, the production of these mutant lines requires an approach targeting simultaneously multiple genes. The CRISPR/Cas9 technique provided such a tool, allowing the mutation of entire gene families with a single transformation event by using multiple gRNAs along with the Cas9 endonuclease (Ordon et al., 2020). In this report, we targeted conserved sequences, shared by multiple LHCB1-coding genes, to design two gRNAs capable of knocking down the five genes. The pale green phenotype and the previously reported lower NPQ level in plants in which LHCB1 was depleted by artificial miRNA (*amiLhcb1*) (Pietrzykowska et al., 2014) facilitated the screening procedure, allowing rapid identification of multiple mutant lines characterized by a complete loss of LHCB1. The selected mutants were all mutated in Chromosome 1 genes (therefore, lacking the Lhcb1.1, Lhcb1.2, and Lhcb1.3 *genes*). Conversely, mutations in *Lhcb1.4* and *Lhcb1.5* CDS were not present in all the lines even if there was no detectable LHCB1 protein. However, it has to be noted that the primary amino acid sequence of the *Lhcb1.4* product is slightly different from the other LHCB1 protein isoforms. This difference, present at the level of the N-terminal portion of the mature protein, may affect the antibody binding and lead to underestimation of the *Lhcb1.4* product (Jansson, 1999). However, the line containing no detectable mutation in the *Lhcb1.4* and *Lhcb1.5* genes, named L1ko “C2a,” did not show any significant difference in Chl content or NPQ when compared to the other L1ko lines (Supplementary Figure 2). This can be explained by a low contribution of *Lhcb1.4* and *Lhcb1.5* to the LHCB1 protein content in our growth condition, despite being reported as relatively highly expressed genes (Jansson, 1999), or by the effect of an undetected mutation outside the CDS that affects these genes.

### Change in Protein Phosphorylation and Photosystem Stoichiometry Partially Compensates for the Loss of LHCB1

Being the most abundant isoform in L1ko lines, the loss of LHCB1 results in the almost complete absence of the trimeric LHCII complex. The differences in antenna and photosynthetic proteins accumulation between L1ko and WT are minor; and, within the expected error of an immunoblot detection, this may hide some significant changes but overall suggests that the mutation caused only limited alterations in terms of protein amount. However, considering the LHCII, the accumulation of LHCB2 increased, while the LHCB3 level showed no significant difference between L1ko and WT. These moderate changes cannot compensate for the LHCB1 loss and align well with the *amiLhcb1* line phenotype (Pietrzykowska et al., 2014). Except for LHCB4, the accumulation of which increased only by around 20% in L1ko compared to WT; the monomeric antennae also show no significant increase. A previous report suggested that the loss of trimeric LHCII was compensated by an increase in the LHCB5 protein (Ruban et al., 2003). LHCB5 was proposed to be integrated into the trimeric LHCII, thanks to the presence of an N-proximal trimerization motif (Hobe et al., 1995). However, a higher accumulation of LHCB5 does not seem to be induced in the L1ko lines as well as in the *amiLhcb1* line (Pietrzykowska et al., 2014) and in the noLHCII mutant (Nicol et al., 2019). Overall, these observations suggest that LHCB5 has no specific role, nor that the accumulation of a specific antenna isoform is induced to compensate the loss of the isoforms composing the LHCII trimers.

Despite similarities between the L1ko mutation *via* CRISPR/Cas9 and the *amiLhcb1* line, differences exist between the two genotypes, notably at the level of STN7 accumulation. In fact, in the *amiLhcb1* line, the amount of the STN7 protein was higher compared to WT (Pietrzykowska et al., 2014). In the mutant lines produced *via* CRISPR/Cas9, we clearly observed the opposite response (Figure 2). The decrease of STN7 in L1ko compared to WT is consistent with the previously reported long-term regulation of STN7 abundance in response to light conditions. Exposure of plants to far-red light, a condition that enhances excitation of PSI over PSII, for a long period, leads to a decrease in the amount of the STN7 kinase (Willig et al., 2011). Therefore, the decrease of STN7 in L1ko can be explained as a consequence of the oxidation of the photosynthetic ETC, as, in the L1ko line, the Chl distribution is shifted in favor of PSI (Figures 3, 4). Conversely, the lower level of TAP38/PPH1, observed in the L1ko lines, is less obvious. In previous reports, the protein levels of STN7 and TAP38/PPH1 were shown to be oppositely regulated; i.e., a decrease of the STN7 kinase was coupled with an increase of the TAP38/PPH1 phosphatase (Wunder et al., 2013). The observation that both STN7 and the counteracting phosphatase are less accumulated in L1ko suggests the existence of another layer of regulation of the protein accumulation, besides the previously documented redox status of the photosynthetic ETC. Consistently, a reduction of the TAP38/PPH1 level was also observed in *amiLhcb1* lines (although to a lesser extent) (Pietrzykowska et al., 2014), which led to the hypothesis that TAP38/PPH1 abundance is also regulated by the presence of its substrate LHCB1 (Suorsa et al., 2016). The observation that the phosphorylation level of the thylakoid protein was lower in L1ko is in agreement with previous reports linking the thylakoid protein phosphorylation to the redox state of the photosynthetic PQ pool more than to the accumulation of the relevant kinases and phosphatases (Suorsa et al., 2016). This appeared to be particularly true for the PSII kinase STN8, as PSBA and PSBC, which are the main targets of this kinase, showed strongly decreased phosphorylation in L1ko compared to WT. However, the lower phosphorylation level was not coupled with a decrease of the relevant kinase, STN8, or the PBCP phosphatase. This supports the hypothesis that their activity is regulated in response to changes in the redox status, previously formulated based on the observation of plants overexpressing the STN8 kinase (Wunder et al., 2013).

### Photosynthetic Electron Transport in L1ko

The clear reduction in STN7 accumulation and phosphorylation of the main thylakoid phospho-proteins in L1ko compared to WT may be related to an imbalance in the redox equilibrium of the photosynthetic ETC. We, therefore, investigated the status of the ETC using Chl fluorescence and transient absorption spectroscopy to detail the impact of the LHCB1 loss on the photosynthetic activity. The first expected impact of the absence of LHCB1, and thus a decrease of LHCII trimers, is a reduction of the PSII physical and functional antenna size (Terao and Katoh, 1996). Consistently, both Chl fluorescence and ECS measurements confirmed that, in L1ko, the maximal PSII photochemical rate measured at a light-limiting intensity of 80-μmol photons m^–2^ s^–1^ was lower in L1ko compared to WT (Figure 3A). Besides the interaction with PSII, several reports have shown that LHCII also contributes to the excitation of PSI (Wientjes et al., 2013; Bos et al., 2017; Chukhutsina et al., 2020). Therefore, we used ECS-based methods to investigate whether the absence of LHCB1 affects the antenna size of PSI. Our data showed that the maximal PSI rate at 80-μmol photons m^–2^ s^–1^ in L1ko is comparable, with a tendency to be larger than that in the WT (Figure 3B). Therefore, no major difference in PSI antenna size should be present between these two genotypes. This can be explained by the relative increase of the LHCA1 and LHCA4 proteins, part of LHCI antenna, over the PSI core (Figure 2). Increased LHCI could compensate for the loss of LHCII, preserving the PSI antenna size. This hypothesis fits well with a previous report showing that an extra LHCI dimer, composed by LHCA1 and LHCA4, can functionally connect to PSI (Crepin et al., 2020). However, both the increase of these LHCI proteins and the decrease of PSI subunits, under our growth conditions, are minimal and thus difficult to confirm by immunodetection. Therefore, we further defined if there was an excitation imbalance between photosystems in L1ko by an indirect measurement of the rate of PQ oxidation by far-red light based on Chl fluorescence rise (Vj) (Toth et al., 2007; Pralon et al., 2020). The result showed that PQ oxidation is faster in L1ko, supporting the idea that the difference between PSI and PSII excitation in far-red light is larger in L1ko compared to the WT background (Figure 3E). The same analysis conducted in the dark showed no difference, suggesting that the difference in the PQ oxidation between L1ko and WT is due to a different relative excitation of PSI and not to an increased contribution of the non-photochemical oxidation of the PQ pool (Supplementary Figure 7; Nawrocki et al., 2015). Consistent with this result, the analysis of the fluorescence decay shows a bigger contribution of the faster component, which is interpreted as the decay of PSI excitation (Wientjes et al., 2017). This confirms that, in L1ko, the global relative Chl pool connected to PSI over PSII is larger compared to the WT; therefore, the decrease in PSII antenna size is not fully compensated by the change in the PSII/PSI reaction center ratio. The increase in the PSII/PSI reaction center ratio is a compensatory response previously observed in Chl *b* mutants as well as in no-LHCII mutants (Terao and Katoh, 1996; Nicol et al., 2019). The PSII/PSI reaction center ratio has also been shown to be adjusted in function of the antenna size by transient induction of Chl *b* synthesis in the Chl *b*-less mutant (Jia et al., 2016). In terms of maintaining the equilibrium of the PQ pool redox state, the excess excitation of PSI could have been partially compensated by an increase of the CEF/LEF ratio. However, there was no significant difference in the CEF over the LEF ratio between the L1ko and WT, suggesting that the CEF/LEF ratio does not play a major role in balancing the ETC redox equilibrium in L1ko (Figure 3F). As the relative PSI/PSII Chl ratio is higher in L1ko than the WT, this should lead to higher oxidation of the photoactive PQ pool under light-limiting condition in the mutant. The oxidation of the photoactive PQ can be inferred by a smaller fraction of closed PSII reaction centers (Krause and Weis, 1984). Consistently, when L1ko plants were analyzed by room temperature fluorescence, we observed a smaller fraction of closed PSII centers as determined by the 1-qL parameter (Kramer et al., 2004), and a higher ΦPSII compared to the WT (Figure 4 and Supplementary Figure 8). As discussed in the previous paragraph, the constitutive over-oxidation of the PQ pool could explain the low phosphorylation of the thylakoid proteins and the low level of STN7 accumulation in L1ko. The qT analysis supports the hypothesis of oxidation of the photosynthetic PQ pool in L1ko. In fact, the changes in the light spectrum that alter the Chl *a* fluorescence, showing a transient peak after the switch from State 1 to State 2 light in WT, have no measurable effect in L1ko (Figure 4). Lack of LHCII caused a low reduction of the PQ pool and, therefore, did not activate STN7 also in the barley *chlorina f2* mutant (Bhalla and Bennett, 1987). This resulted in no measurable quenching related to state transition in this mutant (Lokstein et al., 1994). Consistently, we observed limited phosphorylation of LHCB2 and a minor change in the antenna connected to PSII and PSI in L1ko, calculated from the maximal fluorescence as qT. The relative increase of LHCB2 abundance, therefore, is not sufficient to compensate for the lack of LHCII heterotrimers in L1ko, as shown also by the lack of a visible PSI-LHCI-LHCII complex in this line by native gel separation (Supplementary Figure 5). These observations are consistent with the previous report showing that both *amiLhcb1* and *amiLhcb2* lines had limited state transitions even if the PQ pool was oxidized in the *amiLhcb1* line and reduced in amiLhcb2 (Pietrzykowska et al., 2014). Besides regulating the qT, STN7 activity has also been shown to regulate the grana stacks diameter (Hepworth et al., 2021). With this regard, the low level and activity of STN7 can also have a role in compensating the smaller grana observed in L1ko (discussed below) and contribute to maintain the equilibrium between LEF and CEF in this mutant as proposed in Hepworth et al. (2021).

The extent of NPQ induction in L1ko is lower than in WT; this phenomenon was observed in the amiLhcb1 line (Pietrzykowska et al., 2014), in the asLhcb2 line, which is lacking both LHCB1 and LHCB2 proteins (Johnson et al., 2008), and in mutants of Chl *b* synthesis such as the *chlorina f2* mutant of barley, which lacks specifically of the fast inducible component qE (Lokstein et al., 1993, 1994) and the *ch1-1* mutant of Arabidopsis (Havaux et al., 2007). This further supports the observation of the important role of LHCII proteins as quenching sites (Dall’Osto et al., 2010). The kinetics of NPQ induction and relaxation are slower in L1ko compared with the WT; this is consistent with previous observations made with the mutants lacking both LHCB1 and LHCB2 (Johnson et al., 2008; Nicol et al., 2019), suggesting that the trimeric LHCII, by its structure or abundance, has an important role in NPQ dynamics.

### Lack of the LHCB1 Isoform Alters the Thylakoid Organization

The trimeric LHCII occupies a large part of the thylakoid membrane surface (Kirchhoff et al., 2002). Therefore, it has been proposed to play a major role in the structure of the thylakoids and, in particular, in the grana stacking (Barber, 1980; Daum et al., 2010; Albanese et al., 2020). However, plants deprived of the two major isoforms, LHCB1 and LHCB2, still have grana stacks (Andersson et al., 2003; Nicol et al., 2019). Mutants of Chl *b* synthesis in Arabidopsis, despite having a strong reduction in LHCII protein content, have been reported to still have stacked grana but with lesser stacks and thus occupying a smaller surface of the chloroplast (Murray and Kohorn, 1991; Ruban et al., 2003; Kim et al., 2009). The grana were present also in *amiLhcb1*, depleted only of LHCB1, but with less grana stacks on average when compared to the WT (Pietrzykowska et al., 2014). Our investigation by TEM found that L1ko contains statistically less stacks per grana compared to WT, and, on average, a smaller diameter (Figure 6). This was, in part, expected as, by losing LHCII, the PSII particles in the grana would be smaller and packed more tightly and thus occupy a smaller area (Goral et al., 2012). LHCII phosphorylation by STN7 was linked to the regulation of the grana diameter as discussed earlier (Hepworth et al., 2021). However, also, the STN8/PBCP kinase and phosphatase were proposed to play a role in regulating grana dynamics. In particular, the mutation of STN8, the kinase mainly involved in the phosphorylation of PSII core protein, was associated with an increase in the grana diameter (Fristedt et al., 2009). Further studies have shown that the CURVATURE 1 (CURT1) protein, which is also phosphorylated in an STN8-dependent fashion, is directly involved in regulating the grana diameter and may be responsible for the change of grana width in the *stn8* mutant (Armbruster et al., 2013; Trotta et al., 2019). Therefore, as for STN7, also, the low level of STN8 activity may have a compensatory function in terms of grana width and stability in the L1ko background.

Although the relationship between the protein composition of the thylakoid membrane and the grana structure has been described in detail, there is a less clear consensus about the role played by the membrane lipids in shaping the thylakoid membrane. In this report, we analyzed the galactolipids present in L1ko, and we found no significant difference with the WT (Supplementary Figure 14). The amount of galactolipids does not follow the decrease of the LHCII proteins thus changing the protein to the lipid ratio in the thylakoids of L1ko. This change may affect not only the ultrastructure of the thylakoids but also the electron transport routes by interfering with the lipid mobility and thus hampering the PQ diffusion between PSII and the cytb6f, antagonizing the acclimation strategies of L1ko discussed in the previous paragraph (Johnson et al., 2014; Tyutereva et al., 2017). Quantification of the carotenoids revealed that, in the L1ko lines, the lutein and violaxanthin/neoxanthin content was lower than in the WT (Supplementary Figure 14). This is not surprising since the large majority of the xanthophylls are expected to be bound to LHCII, while most of the β-carotene, which is not decreasing in L1ko compared to WT, is associated with the photosystems core and, mostly, to PSI (Qin et al., 2015). The same decrease in xanthophyll content was not observed in the *asLhcb2* line, which is lacking both LHCB1 and LHCB2 proteins (Andersson et al., 2003), while the carotenoid content decreased to the same extent in the crossed *amiLhcb1*/*amiLhcb2* line (Nicol et al., 2019). A similar response is observed in the Arabidopsis Chl *b* mutant *ch1-3* where, on a Chl *a* basis, the β-carotene content increased, while the xanthophylls lutein and neoxanthin were less accumulated (Kim et al., 2009). Hinting that, in these lines, a signaling mechanism is in place to coordinate the carotenoid metabolism with the antenna biosynthesis (Li et al., 2009).

## Conclusion

In conclusion, we report that the stable loss of LHCB1 induces compensatory mechanisms in the plant. These mechanisms include the alteration of the kinase and phosphatases, regulating the equilibrium of the photosynthetic ETC. Taken together, these data show that loss of LHCB1 cannot be compensated by the other LHCII isoforms, and that this isoform, by its nature or level of accumulation, has a unique role in shaping the thylakoid membrane and maintaining the equilibrium between the two photosystems. Besides, this mutant gives the first possibility for a mutational analysis on the major LHCII, something that, up until now, has been impossible due to the clustered gene organization. Since trimeric LHCII is essential for the plasticity of the photosynthetic machinery that allows the rapid acclimation to changing light conditions, these mutants are not only of vital importance for fundamental research but also for applied projects aiming at increasing crop productivity by improving the light use efficiency.

## Data Availability Statement

The raw data and the pictures used for this manuscript are available at https://zenodo.org/record/5729177#.YaDZILrjKUk. Further data, and the described lines, can be provided by the authors upon reasonable request.

## Author Contributions

HS and FL designed the experimental plan and performed the other experiments. RC, WN, and CH measured the photochemical rate of photosystems. GF and DT performed the CEF/LEF analysis. HS and VD produced the electron microscopy pictures. EW analyzed the images. EW and CS performed confocal microscopy and FLIM analysis. GG performed the lipid profile analysis. FL performed the statistical analysis of the data. All authors contributed in the redaction, read, and approved the manuscript.

## Conflict of Interest

The authors declare that the research was conducted in the absence of any commercial or financial relationships that could be construed as a potential conflict of interest.

## Publisher’s Note

All claims expressed in this article are solely those of the authors and do not necessarily represent those of their affiliated organizations, or those of the publisher, the editors and the reviewers. Any product that may be evaluated in this article, or claim that may be made by its manufacturer, is not guaranteed or endorsed by the publisher.
